# Post-ERCP Pancreatitis: Risk factors and role of NSAIDs in primary prophylaxis

**DOI:** 10.12669/pjms.36.3.1804

**Published:** 2020

**Authors:** Muhammad Haseeb Nawaz, Shahid Sarwar, Muhammad Arif Nadeem

**Affiliations:** 1 Dr. Muhammad Haseeb Nawaz, MBBS, Post Graduate Resident, Medical Unit-III, Department of Medicine & Gastroenterology, Services Institute of Medical Sciences, Lahore, Pakistan; 2 Dr. Shahid Sarwar, MBBS, FCPS (Med), FCPS (Gastroenterol), MCPS-HPE, FRCP (Edin) Associate Professor, Medical Unit-III, Department of Medicine & Gastroenterology, Services Institute of Medical Sciences, Lahore, Pakistan; 3 Prof. Dr. Muhammad Arif Nadeem MBBS, FCPS (Medicine), Medical Unit-III, Department of Medicine & Gastroenterology, Services Institute of Medical Sciences, Lahore, Pakistan

**Keywords:** NSAIDs, Post-ERCP pancreatitis, Primary prophylaxis, Risk factors

## Abstract

**Objective:**

To determine efficacy of diclofenac suppository in reducing post-ERCP pancreatitis (PEP) and identify risk factors for PEP.

**Methods:**

This is a placebo-based prospective study at Department of Medicine & Gastroenterology, Services Institute of Medical Sciences / Services Hospital, Lahore performed from January 2018 to June 2019. Patients were randomized to receive diclofenac suppository or glycerine suppository before ERCP. Both groups were compared for PEP using chi square x^2^ test while risk factors for PEP were determined using binary logistic regression.

**Results:**

Total of 165 patients with mean age 49.1(±15.2) and male to female ratio 1/1.6 (63/102) were included. Among 82 (49.7%) patients in diclofenac group, 8 (9.7%) developed pancreatitis while 19(22.9%) of 83(50.3%) in placebo group had PEP (p value 0.02). After multivariate analysis, age>45 years (p value 0.014, OR 3.2), Bilirubin >3 mg/dl (p value 0.004 OR 3.58), time to cannulation> 5 minutes (p value<0.000 OR 9.2), use of precut (p value< 0.000 OR 4.9), pancreatic duct cannulation (p value 0.000 OR 5.46) and total procedure time >30 minutes (p value 0.01 OR 3.92) were risk factors for PEP.

**Conclusion:**

Pre-procedure Diclofenac suppository reduces post-ERCP pancreatitis. Age > 45 years, serum bilirubin > 3 mg/dl, cannulation time > 5 minutes, use of precut, pancreatic duct cannulation and procedure time > 30 minutes are risk factors for post-ERCP pancreatitis.

## INTRODUCTION

Endoscopic Retrograde Cholangiopancreatography (ERCP), since its inception in 1968 has revolutionized the management of pancreatico-biliary diseases.^1^ It allows access to biliary and pancreatic ducts for diagnosis as well as management without risks of prolonged anesthesia, surgical trauma, surgical complications and extended hospital stay for post-operative recovery with technically difficult pancreatico-biliary surgeries.^2^

In last few decades, with availability of sophisticated new accessories of ERCP, range of therapeutic interventions possible in ERCP has remarkably extended. However, this has resulted in increasing incidence of complications like post ERCP pancreatitis, cholangitis, iatrogenic bleeding, sepsis, perforation etc.^3^ Complications like hypoxia, aspiration pneumonia and cardio-pulmonary depression are increasing due to extended anesthesia time during these therapeutic interventions.^4^

Among all potential complications of ERCP, post ERCP pancreatitis (PEP) is a common complication associated with significant morbidity and mortality.^5^ Its incidence varies between 1-40% depending on patient related co-morbidities, type of intervention, duration of procedure and expertise of endoscopist.^6^ PEP is mostly self-limiting responding to conservative treatment but still caries mortality of 0.7%.^6^

Post ERCP pancreatitis is the result of mechanical, thermal, chemical, enzymatic or hydrostatic injury inflicted during procedure. Based on this pathophysiology, different interventions have been tried for prophylaxis of PEP.^7^ It includes ensuring pancreatic drainage via stenting, inhibition of intra-acinar trypsinogen activation via protease inhibitors, reducing sphincter of oddi spasm with glucagon or glyceryl trinitrate (GTN) and facilitation of cannulation with secretin injection.^8^ However most promising results are shown by anti-inflammatory drugs targeting chemical injury induced cascade of inflammation.^9^

Non-steroidal anti-inflammatory drugs (NSAIDs) are inexpensive, easily administered and effective inhibitors of phospholipase A2 and cyclooxygenase which can block inflammation leading to acute pancreatitis.^9^ Anti-inflammatory drugs evaluated for their efficacy in preventing PEP include indomethacin, celecoxib and diclofenac sodium with mixed results. In a study of 602 patients by Elmunzer et al, reduction in incidence of PEP from 16.9% to 9.2% was noted with use of indomethacin.^10^ Otsuka et al. noted reduction in PEP from 18.9% to 3.9% with use of rectal diclofenac suppository before ERCP.^11^ However no benefit of administering indomethacin in controlling PEP was seen in a study of 665 patients by Dobronte et al.^12^ It is due to these conflicting results that despite recommendation by European Society of Gastrointestinal Endoscopy (ESGE) to use NSAIDs for PEP^13^, its role is still under intense debate. We planned a placebo based, case control study to determine efficacy of NSAIDs (Diclofenac) suppository in preventing post ERCP pancreatitis and to identify risk factors predisposing to PEP.

## METHODS

A quasi-Experimental, placebo based case control, triple blind study was carried out at Department of Medicine & Gastroenterology, Services Institute of Medical Sciences / Services Hospital, Lahore from January 2018 to June 2019 after the approval of Internal Review Board (Ref No. IRB/2018/464/SIMS, dated Sept. 25, 2018). All patients aged above 18 years being admitted for ERCP willing to participate in study were included after informed consent. Patients with allergy to NSAIDs, contraindication for NSAIDs use (i.e. active peptic ulcer disease, serum creatinine> 1.4mg/dl), history of pancreatitis within last 4 weeks, use of NSAIDs in preceding two weeks, antibiotic use within 4 weeks and pregnant and nursing mothers were excluded from study.

Detailed clinical interview regarding symptoms, indication for procedure and co-morbid issue followed by clinical examination was carried out. Laboratory and radiological investigation results including complete blood count, liver function tests, renal function tests, abdominal ultrasound, CT scan, MRI or MRCP if performed were recorded. Patients were randomized in two groups using online random table generator stat trek®. Patients of Group-A were given Diclofenac sodium suppository by nursing assistant at least 15 minutes before procedure while Group-B patient received Glycerine suppository as placebo. Identity of patient group was not known to patient, endoscopist and team responsible for patient follow up after procedure.

All ERCP procedures were performed by two senior endoscopists, under Propofol sedation by a dedicated senior nursing assistant with continuous monitoring of vital signs. All maneuvers and interventions done during ERCP including time of biliary cannulation which was always wire guided, use of needle knife sphincterotomy, pancreatic duct cannulation/contrast injection, sphincterotomy/sphincteroplasty, balloon sweep for stone extraction, biliary or pancreatic stenting and total duration of procedure were recorded.

After ERCP, patients were kept in high dependency unit (HDU) for at least 24 hours. Patients were monitored for new onset abdominal symptoms including pain, vomiting, distention or absolute constipation. Serum amylase/lipase were checked at six hours and 24 hours’ post-procedure. Primary study end point was post-ERCP pancreatitis which was defined and staged according to Atlanta criteria which defines PEP as presence of at least 2 of 3 features including;

(1) Abdominal pain consistent with acute pancreatitis
(2) At least 3 times increase in serum amylase or lipase
(3) Evidence of pancreatic inflammation on abdominal ultrasound, CT scan or MRI.^14^

Diclofenac sodium was considered effective if incidence of PEP decline by >50%. Secondary end points were risk factors associated with PEP. Post procedure follow up was done by senior team members, unaware of treatment given as prophylaxis for PEP. Patients with PEP were managed as per standard protocols for managing acute pancreatitis.

### Statistical Analysis

We estimated that a sample size of 164 will give 80% power to detect at least 50% reduction in incidence of PEP (i.e. from 20% to 10%) with 5% margin of error. Data was analyzed using SPSS 22^®^ (Armonk NY: IBM corp.) by statistician unaware of drugs used in two groups. Quantitative variables with normal distribution were expressed as mean± standard deviation (SD), nonparametric variables were given as median ± interquartile range (IR) whereas qualitative variables were given as percentage. Primary and secondary outcome variables were compared between two groups using unpaired student’s t test and chi square (X^2^) test to determine Odd’s ratio (OR) for PEP and Mann Whitney U test for non-parametric variables.

Cut off values for numerical variables for predicting PEP were determined using Receiver operating characteristic (ROC) curve identifying coordinate point with best sensitivity and specificity. A multi-variate binary logistic regression analysis was performed for variables with statistical significance on uni-variate analysis (p ≤0.05) using post-ERCP pancreatitis as dependent variable. Predictive value of model was checked by determining two log likelihood and testing with Hosmer and Lemeshow test. P value of less than 0.05 was considered statistically significant.

## RESULTS

Total of 165 patients were included with mean age of 49.1 (±15.2) and male/female ratio of 1/1.6 (63/102). Predominant presenting complaints in patients included were abdominal pain 131 (79.4%), jaundice 101 (61.2%), fever 79 (47.9%), itching 78 (47.3%) and weight loss in 66 (40%) patients. Previous history of cholecystectomy was present in 28 (17%) patients, who were undergoing ERCP for residual stones 21(75%) and iatrogenic CBD injury seven (25%). Diabetes mellitus was present in 32 (19.4%) patients while 43 (26.1%) were hypertensive.

Majority of patients were being treated for common bile duct (CBD) stones 97 (58.8%) whereas pancreatic carcinoma 17 (10.3%), cholangiocarcinoma 11 (6.7%), periampullary cancer 11 (6.7%), gall bladder CA seven (4.2%) and CBD leakage 10 (6.1%) were other major indications for ERCP.

Randomization lead to 82 (49.6%) patients in study group receiving Diclofenac suppository before procedure while 83 (50.4%) patients in placebo group, treated with glycerine suppository. We compared both groups for baseline variables as shown in Table-I.

**Table-I:** Comparison of patients in study group and placebo group.

Variable	Study group (Diclofenac suppository) (n-82)	Placebo group (Glycerine Suppository) (n-83)	P-value
Age (mean years ± SD)	47.7± (14.8)	50.2 ±(15.5)	0.28
Duration of illness (median ± IQR)	122 ±(189)	116 ±(159)	0.81*
Serum bilirubin (median ± IQR)	7 ±(8)	7 ±(9)	0.15*
Male/Female (number)	28/54	35/48	0.28
H/O Surgery	15	13	0.65
Diabetes mellitus	15	17	0.72
Hypertension	21	22	0.89
CBD stone	51	46	0.37
Malignancy Cholangio/pancreatic/Gall bladder	8/21/6	14/17/1	0.12
CBD leakage	5	5	0.60

* Mann Whittney U-test, IQR: Interquartile Range.

During ERCP, cannulation was achieved within five minutes in 89 (53.9%) patient, needle knife sphincterotomy (precut) was needed in 28(17%) patients while 131 (47.3%) had routine sphincterotomy after cannulation. Pancreatic duct (PD) was cannulated in 45 (27.3%) patients and 10 (6.1%) had contrast injection in PD as well. CBD stones were extracted in 83 (50.3%) patients while 78 (47.3%) had biliary stenting and 10 (6.1%) had CBD dilatation.

After ERCP, 57 (34.5%) patients complained of abdominal pain, 25 (15.2%) had vomiting and 16 (9.7%) felt abdominal distension. These complaints settled in majority of these patients within few hours. Post-ERCP pancreatitis (PEP) was diagnosed in 27 (16.4%) patients, all of whom had mild pancreatitis and recovered in few days without necrosis or multi-organ failure. Asymptomatic hyper-amylasemia was seen in 13 (7.9%) patients. PEP was significantly more in placebo group 19 (22.9%) patients as compared to eight (9.7%) patients in study group receiving diclofenac suppository (p value 0.02) confirming its efficacy with >50% reduction in PEP with Odds ratio (OR) of 0.36 (95% CI: 0.14-0.88) in favor of diclofenac group.

**Fig.1:**
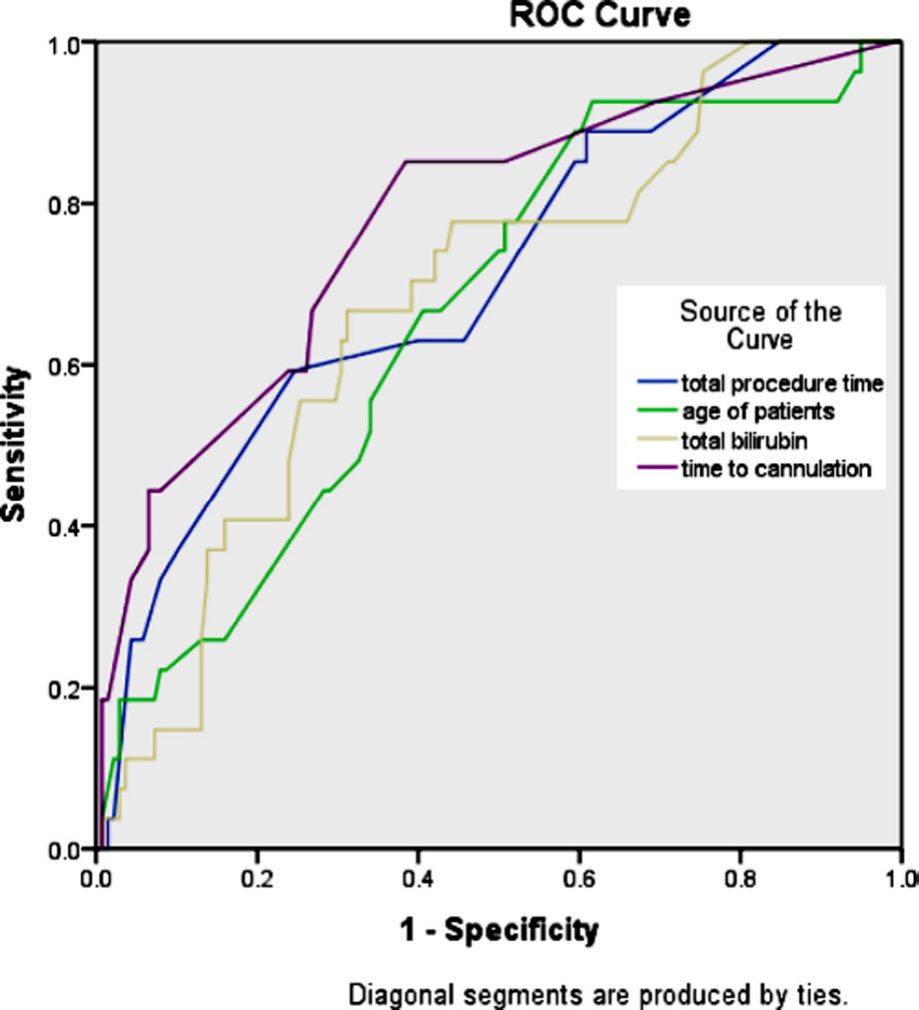
ROC Curve to identify best coordinate points.

On uni-variate analysis of variables for its association with PEP, we identified age>45 years (p value 0.014, OR 3.2 95% CI:1.2-8.4), Bilirubin >3 mg/dl (p value 0.004, OR 3.58 95% CI: 1.4-8.7), time of cannulation(TTC) > 5 minutes (p value<0.000, OR 9.2 95%CI:3.0-28.1), use of needle knife for cannulation(precut) (p value<0.000, OR 4.9 95% CI: 1.9-12.2), pancreatic duct cannulation (p-value<0.000, OR 5.46 95% CI: 2.2-13) and total procedure time (TPT) >30 minutes (p value 0.01, OR 3.92 95% CI:1.2-11.9) as significant risk factors for PEP. However, patients undergoing ERCP for CBD stones had significantly less chance of developing PEP (p value 0.003, OR 0.28 95% CI 0.11-0.68). ROC curves to identify cut off values of age, bilirubin, TTC and TPT are shown in Fig.1.

The test result variable(s): total procedure time, age of patients, total bilirubin and time to cannulation has at least one tie between the positive actual state group and the negative actual state group. Statistics may be biased.

We analyzed prediction model comprising of age> 45, serum bilirubin > 3 mg/dl, time to cannulation (TTC) > 5 minutes, precut, pancreatic duct cannulation and total procedure time (TPT)>30 minutes via binary logistic regression. Our model accurately predicted chances of PEP in 86.1% cases (p-value of <0.000) with 2 log likelihood ratio of 114.87. Hosmer and Lameshow goodness of fit testing revealed no difference in model based expected outcome and observed outcome (p-value 0.38) as shown in Table-II and Table-III.

**Table-II:** Hosmer and Lameshow goodness of fit testing of model for predicting PEP.

Contingency Table for Hosmer and Lemeshow Test.						

		Clinical pancreatitis = No		Clinical pancreatitis = Yes		Total

		Observed	Expected	Observed	Expected	
Step 1	1	12	11.771	0	0.229	12
	2	19	18.632	0	0.368	19
	3	12	12.472	1	0.528	13
	4	13	12.463	0	0.537	13
	5	14	16.117	3	0.883	17
	6	13	11.882	0	1.118	13
	7	16	16.325	3	2.675	19
	8	16	15.938	4	4.062	20
	9	14	12.916	6	7.084	20
	10	9	9.483	10	9.517	19

*Hosmer and Lameshow Test.*						

	*Step*	*Chi-square*		*df*	*Sig.*	

	1	8.544		8	0.382	

**Table-III:** Classification Tablea.

	Observed		Predicted		

			Clinical pancreatitis		Percentage Correct

			No	Yes	
Step 1	Clinical pancreatitis	No	134	4	97.1
		Yes	19	8	29.6
	Overall Percentage				86.1

## DISCUSSION

Despite lot of technological advances in endoscopy, post-ERCP pancreatitis continues to be a major complication encountered with incidence varying from 1% to 40% depending on patient as well as procedure related risk factors. Syren E et al. in a retrospective analysis identified female gender, age below 65 years and hyperlipidemia as potential risk factors for PEP.^15^ More than 10 attempts at cannulation (OR 14.9), previous PEP (OR 8.7), precut (OR 3.1), pancreatic duct cannulation (OR 2.1) were identified as risk factor for PEP in a prospective multicenter study.^16^ Li GZ et al in a study of 1786 ERCPs, noted 3.8% incidence of PEP and identified pancreatic deep wire pass, metal biliary endoprosthesis, post liver transplantation and post-fistulotomy ERCP as risk factors of PEP.^17^ Suspected sphincter of Oddi dysfunction, presence of hilar obstruction, number of cannulation attempts>13, pancreatic duct cannulation≥1 and pancreatic contrast injections≥1 were potential risk factors for PEP in a study of 790 patients by Kang X et al.^18^ Difficult and prolonged cannulation (p 0.002), pancreatic duct cannulation (p 0.001) and pancreatic duct contrast injection (p<0.001) were associated with PEP in a study from Karachi.^19^

We in our study identified two patient related risk factors, age >45 years and bilirubin >3 mg/dl and four procedure related factors time taken for cannulation>5 minutes, use of needle knife, pancreatic duct cannulation and procedure time >30 minutes to be associated with post-ERCP pancreatitis. Prolonged cannulation time, pancreatic duct cannulation and needle knife use results in mechanical and thermal injury increasing risk of inducing cascade of pancreatic inflammation. Synergy of these risk factors increases chances of PEP^13^ as verified by 86.4% accuracy in predicting PEP in our study when these risk factors are combined. Due care for indication of procedure, improved technical skills with avoidance of hazardous interventions like pre-cut or unintended pancreatic duct cannulation and efficient procedure time can reduce chances of PEP.

We diagnosed PEP in 16.4% study patients, significantly less (9.7%) in study group treated with Diclofenac suppository before procedure than placebo group (22.9%). Out of all interventions tried to avoid PEP, NSAIDs use has shown best results. In a meta-analysis of 19 RCT involving 5031 patients NSAIDs use was associated with significant PEP risk reduction (RR=0.45, 95% ci 0.30 to 0.67).^20^ Serrano JPR did a systematic review of 21 RCTs comprising of 6854 patients comparing NSAIDs vs placebo before ERCP and concluded that only rectal administration reduces incidence of PEP (6.8% VS 13%; 95% CI 0.10-0.04, Numbers needed to treat (NNT) 20, P <0.05). Moreover, only diclofenac and indomethacin were effective in preventing PEP.^21^ It is due to this robust evidence that ESGE has recommended routine use of rectal NSAIDs before every ERCP.^13^

Despite recent recommendation by ESGE, use of NSAIDs for PEP prophylaxis is not common in clinical practice. In a recent survey from Portugal, only 54% patients undergoing ERCP received rectal NSAIDs.^22^ Similarly 64.1% of PEP prophylaxis non users cited lack of conviction in its benefit for their decision in a survey from UK.^23^ Studies like ours depicting clear benefit of using NSAIDs for preventing PEP will promote its use in clinical practice leading to significant reduction in potentially lethal complication like PEP.

## CONCLUSION

Pre-procedure Diclofenac suppository reduces post-ERCP pancreatitis. Age> 45 years, serum bilirubin> 3 mg/dl, cannulation time in excess of 5 minutes, use of precut, pancreatic duct cannulation and procedure time > 30 minutes are risk factors for post-ERCP pancreatitis.

### Author’s contributions:

**MHN:** Designed study, data collection, manuscript review.

**SS:** Conceived and designed study, statistical analysis, manuscript writing, is responsible for integrity of research.

**MAN:** Designed study, Review and final approval of manuscript.
